# Epidemiological and economic consequences of lumpy skin disease outbreaks on farm households in Khyber Pakhtunkhwa, Pakistan

**DOI:** 10.3389/fvets.2023.1238771

**Published:** 2023-12-21

**Authors:** Shahab E. Saqib, Muhammad Yaseen, Supawan Visetnoi, Sikandar, Shoukat Ali

**Affiliations:** ^1^School of Agricultural Resources, Chulalongkorn University, Bangkok, Thailand; ^2^Abdul Wali Khan University Mardan, Mardan, Khyber Pakhtunkhwa, Pakistan; ^3^Institute of Agricultural Extension, Education and Rural Development, University of Agriculture Faisalabad, Faisalabad, Pakistan

**Keywords:** farm households, livestock, economic losses, economic effect, livelihood impact

## Abstract

The livestock sector plays a crucial role in sustaining the livelihoods of millions of families across the world, especially in developing countries. However, farming households that rely on agriculture and livestock are particularly susceptible to the impacts of various infectious diseases and natural disasters. This study focuses on estimating the economic burden imposed on households by lumpy skin disease (LSD) in Pakistan and explores the effect of various socioeconomic factors on mortality ratio. Data were collected through a questionnaire survey from 406 farmers and were analyzed through descriptive statistics to calculate the monetary losses. In addition, the study employed fractional probit regression to identify factors affecting mortality ratio. The results demonstrate significant economic impacts of LSD on farm households in Pakistan, leading to direct and indirect losses and reduced milk productivity. Exotic cows were found to be more susceptible to mortality compared to indigenous cows. The study also found that farmers’ education, experience, household income per month, vaccination, domestic-commercial, commercial animals, and access to information were negatively associated with mortality. The findings of this study emphasize the need for preventative measures such as affordable vaccines, treatment, and improved livestock health and welfare to mitigate the negative effects of LSD on farmers’ income and the local economy.

## 1 Introduction

In Pakistan, the livestock sector is an important source of income for 8 million of families and receive 35–40% of their earnings from this sector (1). According to Pakistan Economic Survey (2) report, livestock farming contributed up to 61.89% of the agriculture sector and 14.04% of the country’s Gross Domestic Product (GDP), showed a growth rate of 3.26% compared to 2.38% in the same period the previous year. Undoubtedly, the livestock sector is important for providing food, income, and employment for many people worldwide (3), however, it also faces a range of risks in the form of various diseases that can affect this sector and its stakeholders. For example, livestock diseases could spread rapidly and cause significant economic losses for farmers and the industry as a whole (4). Lumpy Skin Disease (LSD) is one of among these diseases that recently caused significant losses to the farmers (5). A large-scale outbreak of LSD has affected the livelihoods of these families. Whereas, for the top 10 countries with the largest buffalo and cattle population (5), LSD was first detected in Pakistan in November 2021 and officially reported by the government on March 4^th^, 2022. Since 2022, the disease has spread widely and has affected farmers mostly in all provinces in Pakistan (6). The ongoing presence of LSD in the country highlights the need for continued monitoring and control efforts to mitigate the impacts of this disease on the livestock sector and the livelihood of those dependent on it (5). In addition, Ullah et al. (7) reported that the LSD is a highly contagious viral disease affecting cows and buffalo worldwide and effective control and management measures are crucial to prevent the spread of the disease and minimize its impact on the livestock sector.

The LSD is a viral infection that affects animals, especially cows and buffalos (5). The primary mechanism of transmission is through arthropod vectors, such as insects; however, there is also a view that direct contact with infected animals, as well as exposure to contaminated feed and water, may contribute to the spread of the virus (8). Since there are no specific antiviral drugs available at this time, the only option for treating lumpy skin disease virus (LSDV) in livestock is to provide supportive care (9). In instances when secondary infections occur, the utilization of nonsteroidal anti-inflammatory medications or antibiotics may be employed as therapeutic interventions (10). The mortality rate associated with lumpy skin disease (LSD) is generally low (1 to 3%), however, a higher morbidity rate (3 to 85%) around the world (11). Studies from Das et al. (12) and Saltykov et al. (13) revealed that LSD caused significant socioeconomic collapses in affected areas. These socioeconomic collapses included in reduced milk production, infertility, and reduction in meat consumption, leading to economic losses for farmers (12). According to FAO report in South East and Southeast Asia, the economic impact of LSD has reached $1.45 billion (14). The LSD had a devastating impact around the world, i.e., the estimated cost for treating LSD in Jordan, including the medication for affected cattle using broad-spectrum antibiotics and anti-inflammatory drugs, was approximately US$ 35.04 per head (12). Moreover, a risk assessment study conducted on the Ethiopian bull market estimated a financial loss of US$ 667,785.6 (15). Moreover, the LSD outbreak in Thailand resulted in economic losses of 68,943 USD throughout the duration of the outbreak. Dairy producers encountered a range of losses, spanning from 8.23 to 9.96 tons of milk each month and their monthly income suffered a decline ranging from 119.43 and 412.57 USD (16). Another study from India reported an estimated impact of USD 2217.26 million (17). In addition, the average amount of economic damage per case was 9,384.41 BDT, which was equivalent to 110.40 USD in Bangladesh (18). Hence, LSD has major consequences for livestock producers as well as for the communities that are directly affected by the disease.

Pakistan has experienced a high number of cases of LSD outbreaks; reported 190,000 cases and it is estimated that more than 7,500 deaths of animal have been occurred due to the illness (8). The LSD has a major impact on cattle production, causing a substantial decrease in milk yield ranging from 10 to 85% (19). Moreover, the spread of LSD has hampered milk and meat sales by 60 to 70%. This resulted in huge economic losses for farmers, highlighting the need for effective control and prevention measures to minimize the impacts of LSD on farmers communities (20). A study conducted by Roche et al. (21) revealed that Pakistan has a large number and density of susceptible cows and buffalo populations which were at high risk. On the other hand, livestock (cows and buffaloes) plays a vital role in the rural economy of Pakistan (22). These animals are an important source of income and livelihood for farm households (22, 23). The LSD outbreak had disrupted the production and productivity of these animals, affecting the farmers’ income and well-being (8). However, there is limited comprehensive data and information available on the economic consequences of LSD outbreaks (24). Therefore, this study aimed to explore and calculate the direct and indirect losses from LSD and to uncover the sources of information to help farmers’ adaptation in the epidemic situation and investigated the factors associated with mortality losses. This study has helped to fill this knowledge gap and provided valuable insights for policymakers and stakeholders. The study findings guided policymakers in formulating appropriate strategies and interventions to mitigate the economic impacts of LSD outbreaks. This included measures to prevent the spread of the disease, improve disease management, and support affected farmers during and after outbreaks. The main objectives of this study are:

1. To examine and quantify both direct and indirect losses caused by LSD outbreaks.
2. To identify the sources of information the farmers need to deal with disease outbreak.
3. To identify and analyses the factors that are associated with mortality ratio of livestock caused by LSD.

The subsequent sections of the study are organized in the following manner: The second section is methods, provided a detailed explanation of the research methods, specifically focusing on the sampling procedure and regression analysis. The third section is the results presented the empirical data obtained from our investigation. The section four is the discussion of results provided an in-depth analysis and contextualization of the findings, establishing connections with existing literature. Within the fifth section, the limitations and strengths of the study are discussed, whereby various constraints encountered and emphasized the important strengths were acknowledged. Finally, inside the conclusion section, the main findings, policy implications and future studies recommendations were discussed.

## 2 Methods

### 2.1 Study area

**Figure 1:**
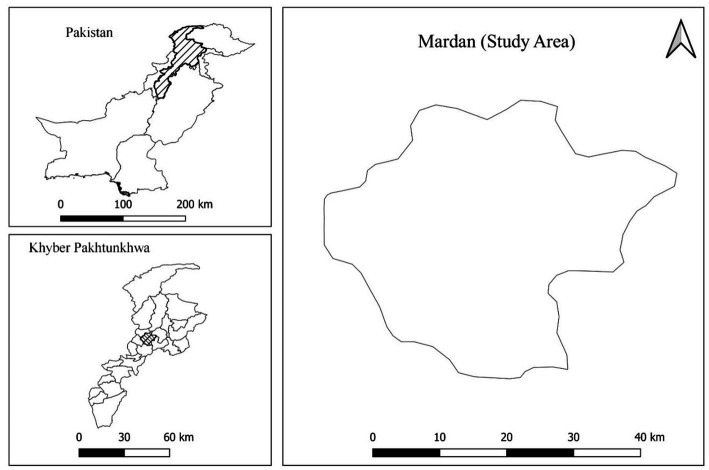
Location map of Mardan.

We have purposively selected Mardan which is the second largest district in Khyber Pakhtunkhwa in terms of farming population and livestock production (25, 26) and is situated in the central zone of the province (Figure 1). Mardan District is located in the center of Khyber Pakhtunkhwa province, and it is bounded by Buner and Malakand districts to the north, Swabi and Buner districts to the east, Nowshera district to the south and Charsadda and Malakand districts to the west. According to the local district office data, Mardan has a total livestock herd population of 0.6 million domestic animals, including 0.2 million cattle, 0.11 million buffaloes, 0.05 million sheep, and 0.20 million goats (27). The overall human population of the Mardan district is 2.1 million people and it covers an area of 1,632 square kilometers (26). Livestock such as cows, buffaloes, goats, and sheep have a significant value in the district’s livelihood, as it is a primary source of income for farmers (KP-BOIT, 2023). They provided revenue through the sale of milk, meat, and other animal products. Cattle are up to39% of the district’s livestock, followed by goats (32%), buffalo (18%), and sheep (8%), with the remaining 3% split between camels, horses, mules, and donkeys and 105 fish farms and 375 poultry farms, all of which are operated by independent entrepreneurs (28).

### 2.2 Sampling

A multistage sampling has been adopted for this study following Andaleeb et al. (29). First, the district Mardan was purposely selected due to the high agricultural activities and livestock population (27). Second, the livestock infected data was collected from district livestock office Mardan. This was secondary data collected by the district government including all details of the animals infected/vaccinated with other households’ information. The data showed that 50,000 farm households were affected by LSD (30). These cases were identified by the presence of distinctive skin lesions, including nodules, swellings, scabs, mainly on the skin of the head, neck, udder of the animals and signs of distress, such as fever, inappetence, and reduced milk production. These lists of farmers were obtained from the office and was properly numbered and entered in Excel. We have followed Saqib et al. (31) and Khan et al. (32) by using the Yamane (33) formula, which is mentioned in Eq. (1), with the procession value set as 5%. The calculated sample size was 397.


(1) $$n=\frac{N}{\left(1+Ne^{2}\right)}$$

*n* = Sample size

*N* = Total number of affected farming households

*e* = Precision value, set as ±5% (0.05)

### 2.3 Data collection

All of the household information was entered to the excel sheets. The original pool of 420 households for interviews was generated at random. Enumerators were employed to conduct door-to-door interviews with these farm households. These enumerators were given a week of intensive training before being closely monitored and corrected as they conducted a survey. The data were collected during January 2023 to March 2023. Keeping in view, if the sample size can be increased beyond what is estimated via statistical formula, the results will be more indicative of the entire population (34), we have increased the survey from 397 to 420 households. Out of 420 questionnaires distributed, 14 incomplete ones were excluded from analysis. Hence, the total sample households which were analyzed in this study were 406 farm households with a response rate of 96.66%. The data were collected through a semi-structured questionnaire administered to livestock farmers in the target area. The questionnaires had two sections, respondent’s personal information, the financial impact of LSD outbreaks on livestock, such as direct monetary losses, reduced productivity, and treatment costs. The indicators for which the data were collected are obtained from literature mentioned in Table 1. The questionnaire was discussed with the directorate of agriculture Khyber Pakhtunkhwa and their suggestion were incorporated. Moreover, an expert from Université de Pau et des Pays de l’Adour has reviewed and edited the questionnaire.

**Table 1:** Variables and their description.

Variables	Description	Source
Age	Age in years	(35)
Education	Years of schooling	(35, 36)
Experience	Experience in years	(35)
Household income	Income per month	(24)
Family members	Number of family members	(37)
Dependent family member	Number of dependent family members	(26)
Domestic	Animals purpose: 1=Domestic, 0=otherwise	(38)
Domestic commercial	Animals purpose: 1=Domestic & Commercial Both, 0=otherwise	(38)
Commercial	Animals purpose: 1= Commercial both, 0=otherwise	(38)
Family type	1=Nuclear, 0=Joint Family	
House Types	1=Kacha House, 0=otherwise	(26)
Vaccination	1=Vaccination used for LSD, 0=otherwise	(15, 39)
Extension services Information	1=Information accessed, 0=otherwise	(15, 39)
Training	1=participated in training, 0=otherwise	(15, 39)
Mortality Loss	The lost measured in PKR due to death of animals	(15, 39)
Direct cost	The out of pocket expenditures on medicine, vaccination	(15, 39)
Indirect cost	The workdays lost of farmer due animal sickness and the loss of milk production	(15, 39)
Mortality ratio	The ratio of animal died due to LSD with total animals	(15, 39)

Source: Authors’ review of literature.

### 2.4 Inclusion and exclusion criteria

In the study, farmers aged 18 years and older who were impacted by LSD were included, while those in other districts of the province or unaffected by LSD were excluded from the study population. Moreover, the cost related to LSD was in the focus of this study. Other diseases costs and mortality were excluded from this study.

### 2.5 Ethical considerations

To conduct this study in the district, the approval was granted by provincial agriculture department of Khyber Pakhtunkhwa, Pakistan. Then, the study was approved by the ethics review committee and the informed consents were obtained from the study participants. The participation in the study was voluntary and participants could withdraw anytime from the study. Moreover, they were informed about the study aims and objectives.

### 2.6 Data analysis

For data analysis, both descriptive and analytical statistical techniques were used. Descriptive statistics such mean, maximum, minimum values, standard deviations were used for the cost analysis. The data were also analyzed using the bar graphs. In analytical statistics, we have used the multivariate fractional probit regression model to determine the factors influencing the mortality ratio.

#### 2.6.1 Study variables

The variables shown in Table 1 contain important demographic and socio-economic characteristics of farm household, including Age (measured in years), Education (years of formal education) and years of experience in farming. The measurement of household income, denoted in monthly earnings in PKR, serves as an indicator of the economic condition of the farm household. Additionally, the variables of family members and dependent family members contribute to the assessment of family’s magnitude and the extent of financial reliance within these households. The domestic category contains animals used for domestic purposes, while the domestic-commercial category denotes animals that serve both domestic and commercial functions. Lastly, the commercial category is having animals exclusively utilized for commercial purposes. The variables of family form and house types are used to classify the family structure (nuclear or joint^1^) and the form of housing (kacha house or pacca house^2^) into distinct categories. Binary variables were used to capture information regarding the use of vaccination in animal care and the access of households to information through extension services. Some information sources that have been used in this study are mentioned in Table 2. These sources included, the use of mobile phone for text messages, social media, TV, radio, friends and relatives. The variable “training” is a binary indicator that signifies whether an individual has participated in training program. This variable has the ability to impact the individual’s degree of knowledge and expertise in the field of animal farming. The dataset comprises of various variables pertaining to financial factors, such as mortality loss (measured in PKR,^3^ representing the economic consequences of animal mortality), direct cost (referring to personal expenditures on medication and vaccination), and indirect cost (indicating the number of workdays missed due to animal illness and decreased milk output). The vaccination for LSD was before the disease infected the animals. The mortality loss was calculated based on the participants responses, later on validated from the market rate. Moreover, the cost of vaccination was obtained directly from the participants. Milk rate data (PKR 200 to 250) was obtained from the surrounding market and then converted the milk loss to monetary term. The workdays lost were multiplied with minimum wage rate (PKR 1000) and loss in monetary term was calculated.

**Table 2:** Descriptive statistics of study variables.

Socioeconomic factors	Min	Max	Mean	SD
Mortality ratio	0.00	1.00	0.481	0.412
Age	28.00	65.00	43.22	8.66
Education	6.00	18.00	9.26	2.26
Household income	14000.00	30000.00	20231.52	2988.13
Income from cattle faming	3000.00	17500.00	6871.67	2995.95
Family members	4.00	25.00	9.61	3.20
Dependent family members	1.00	8.00	1.96	1.24

Type of house	*f*	%	–
Kacha house	253	62.3	–
Pacca house	153	37.7	–
Family type	–	–	–
Joint family	235	57.9	–
Nuclear family	171	42.1	–
Training	104	25.9	–
Extension services	122	30.3	–

Sources of information			Ranks
Mobile phone	250	61.6	I
Social media	402	99	II
TV	241	59.4	III
Radio	277	68.2	IV
Friends	121	29.8	V
Relatives	100	24.6	VI
I do not Know	26	6.4	VII

Source: Field survey.

#### 2.6.2 Cost analysis

The mortality loss was calculated based on the participants responses and estimated by using Eq. (2).


(2) $$ML=n_{ij}\times MR_{ij}$$

ML is the motility loss in monetary terms, n is number of animals died due to LSD, i represents the ith household, j shows the bread of animal either exotic or domestic and MR is the market rate of the animal died.

For the cost estimation of vaccination, we have used Eq. (3).


(3) $$VC=a_{i}\times t\times x_{i}$$

Where, VC is the vaccination cost, a represents the number of animals, t is number of vaccination, and x is the household. Likewise, the milk rate data was obtained from the surrounding markets and then converted the milk loss to monetary loss using Eq. (4).


(4) $$MLC=m_{i}\times milR$$

MLC is the milk loss, $m_{i}$ shows the number of litters of milk loss per ith household and milR is the milk rate per liter.

The workdays lost was monetarized using Eq. (5) below:


(5) $$WL=D_{k}\times L\times wageR$$

WL is for the cost from the work days lost, $D_{k}$ is the number of days by the k labor, L shows the proportion of days lost and wageR represents the wage rate.

#### 2.6.3 Regression model

The study has used multivariate fractional probit model which is a statistical model used to analyze and model data with a dependent variable that typically represents proportions, probabilities, or values between 0 and 1 (40). In the fractional probit model, it is presumed that the dependent variable follows a fractional distribution, typically with a mean between 0 and 1 (41). The fractional probit model is defined as follows in Eq. (6):


(6) $$y=\alpha_{i}+X_{i}\beta_{i}+\varepsilon_{i}$$

y is a dependent variable that was the mortality ratio, its value ranges from 0 to 1. Where $X_{i}\;$ is the 1×k vector of observed independent variables and $\beta_{i}$ is the k×1 vector of the unknown parameters and $\varepsilon_{i}$ represents the error term. The dependent variable ( y) is calculated by Eq. (7), below:


(7) $$y=\frac{Z_{i}}{A_{i}}$$

Where $Z_{i}$ are the number of animals died due to LSD in the ith household and $A_{i}$ represents the total number of animals. The variables were checked for correlation and those variables were used in the model having significant relationship with dependent variable. Moreover, the independent variables were checked for the multicollinearity, which resulted no multicollinearity.

## 3 Results

### 3.1 Socio-economic characteristics of farm households

The results presented in Table 2 show that mean value of mortality ratio was 0.481. It implies that nearly half of the animals died due to LSD per household. Moreover, the farmers’ ages range from 28 to 65 years old, with a mean age of 43.22 years and a standard deviation of 8.66. The second variable was education, which measured the number of years of schooling completed by the farmers. It is reported that the range of education levels was from 6 to 18 years of schooling, with a mean of 9.26 years and a standard deviation of 2.26. The third variable was household income, which measured the monthly income of the farmers. It is revealed that the range of households’ incomes was from PKR 14,000 to 30,000, with a mean income of 20,231.52. The income from cattle farming showed that mean income was PKR 6,871.67. It implied that 34% of the monthly income came from cattle farming within these households. Moreover, family members measured the number of family members living with the farmer. It showed that the range of family members was from 4 to 25, with a mean of 9.61 and a standard deviation of 3.20. The results for dependent family members measured the number of dependents living with farmer. It is found that the range of dependents was from 1 to 8, with a mean of 1.96 and a standard deviation of 1.24. Moreover, results in Table 1 shows that 253 participants (62.3%) live in Kacha houses, while 153 participants (37.7%) live in Pacca houses, and most of them were living in joint families (57.9%). The most common source of information among the respondents is social media, with 402 participants (99%) using it. The second most common source is mobile phones, with 250 participants (61.6%) using it. TV is the third most common source, with 241 participants (59.4%) using it, followed by radio with 277 participants (68.2%). Friends are the fifth most common source of information, with 121 participants (29.8%) using it.

### 3.2 Farmers’ knowledge about the causes of lumpy skin disease and sources of information helped in adaptation

**Figure 2:**
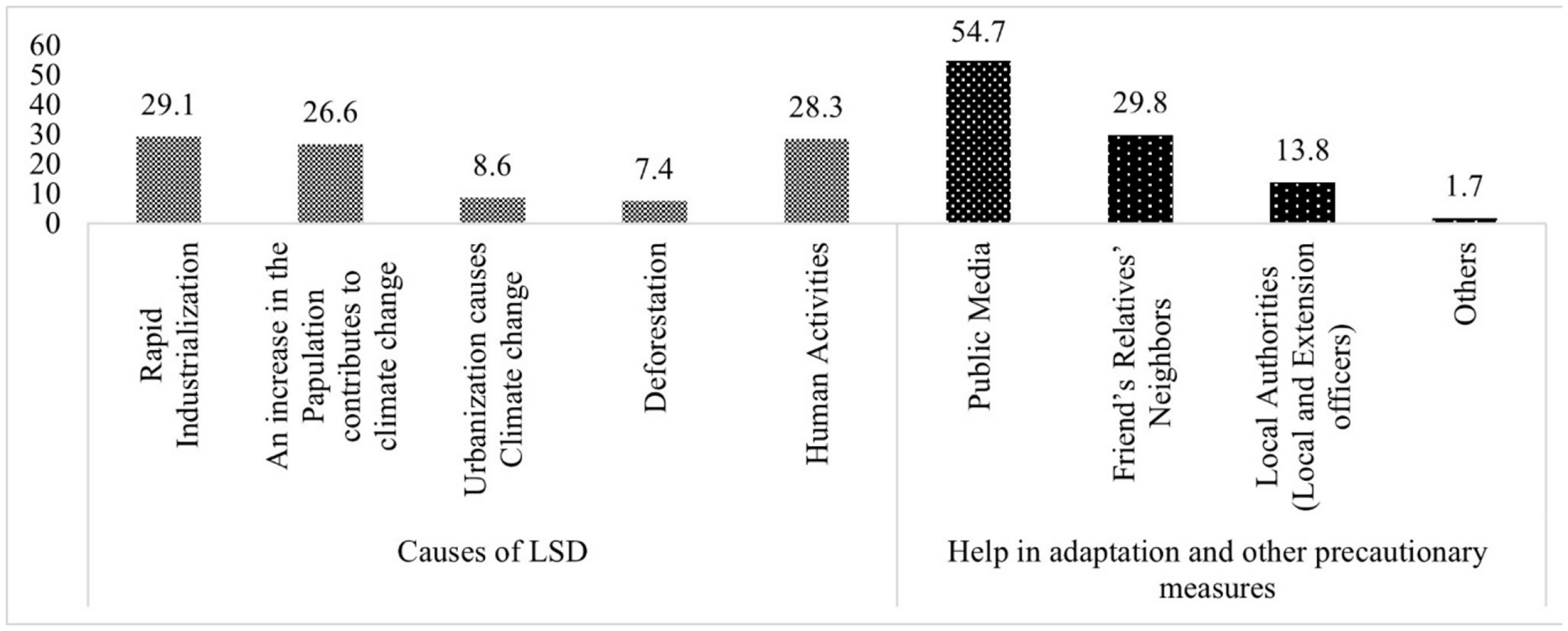
Causes and sources of precautionary measures of LSD.

The Figure 2 shows farmers’ perceptions of the causes of LSD and their sources of information on how to adapt to it. About 29.1% of farmers identified rapid industrialization as the cause of LSD and26.6% of the farmers mentioned the increase in population as a contributing factor. While, 8.6% of the farmers believed that urbanization is a cause of LSD. The figure below also provided the sources of information that farmers relied on how to adapt to this disease. Public media was identified as the main source of information (54.7%) that helped farmers in adaptation measures and 29.8% of farmers were helped and informed by friends, relatives, and neighbors.

### 3.3 Economic losses from LSD

The statistics for mortality losses in indigenous cattle show the loss ranges from 0.00 to 14.00, with a mean of 3.37 (Table 3). Mortality losses indigenous breed PKR: This statistic shows the range of monetary losses in Pakistani Rupees (PKR) due to the mortality of indigenous cattle. The range is from 0.00 to 480,000.00 PKR, with a mean of 132,906.40 PKR. Mortality losses of exotic breed due to LSD is from 0.00 to 20.00, with a mean of 12.7562, while its loss in PKR shows a mean of 860,467.98 PKR. The total loss statistics show that both indigenous cattle and exotic cows range from 0.00 to 4,230,000 PKR. The LSD is associated with both direct and indirect costs, including veterinary expenses, decreased productivity, and financial losses for owners. The farmers reported direct cost that included PKR.175.84 per animal on account of vaccination costs for each dose and the treatment cost which was the highest PKR. 6869.69 per animal on average. The indirect cost included milk loss and work days loss of the farmer. The results show that farmers work days lost that were converted into monetary value, having an average value of 2,222.66 PKR. If the animal with lumpy skin is used for breeding or milking, it can have a negative impact on human health, that caused milk productivity loss which was PKR 17,280.

**Table 3:** Impact of LSD.

Description	Min	Max	Mean	SD
Impact on livestock assets				
Buffalo	0.00	40.00	3.24	5.25
Cow	0.00	38.00	3.57	5.04
Infected animals	0.00	25.00	2.89	2.62
Total no of animals	1.00	95.00	15.5123	6.00
Mortality losses indigenous breed	0.00	14.00	3.37	2.09
Mortality losses indigenous breed PKR	0.00	480000.00	132906.40	75076.42
Mortality losses exotic breed	0.00	20.00	12.7562	16.02
Mortality losses exotic breed PKR	0.00	3825000	860467.98	452112.35
Total mortality losses PKR	0.00	4230000	993374.38	495952.32
Direct cost				
Vaccination cost PKR/Animal	98.08	230.00	175.84	9.48
Treatment cost PKR/Animal	0.00	6966.14	6869.69	651.17
Indirect cost				
Wage loss (PKR)	800	9,600	2222.66	985.06
Milk loss PKR	640.00	17280.00	2037.43	1878.99

Source: Authors’ calculation.

### 3.4 Results of correlation

The provided correlation matrix (Table 4) shows the relationships between independent variables and the mortality ratio. The results show that all the variables were significant in relationship except dependent family members and family type. Hence, these two variables were excluded from the final model. There are negative correlations between the mortality ratio and variables such as education level, experience, household income, household size, domestic-commercial animals, commercial animals, house type, vaccination, access to extension services for information and training. However, age of the participants, domestic animals show positive relationship with motility ratio.

**Table 4:** Correlation matrix of the study variables.

Variables	Y	X_1_	X_2_	X_3_	X_4_	X_5_	X_6_	X_7_	X_8_	X_9_	X_10_	X_11_	X_12_	X_13_	X_14_
Mortality Ratio (Y)	1																											
Age (X_1_)	0.133^**^	1																									
Education (X_2_)	−0.275^***^	−0.284^**^	1																							
Experience (X_3_)	−0.209^**^	0.838^**^	−0.337^**^	1																					
Household income (X_4_)	−0.117^**^	0.013	0.099^*^	0.090^*^	1																			
Household size (X_5_)	−0.242^*^	−0.010	0.232^**^	−0.031	0.434^**^	1																	
Dependent family member (X_6_)	−0.111	0.003	0.161^**^	−0.061	−0.042	0.115^**^	1															
Domestic (X_7_)	0.089^*^	−0.031	0.125^**^	−0.032	0.026	0.164^**^	0.073	1													
Domestic-commercial (X_8_)	−0.111^***^	0.003	0.161^**^	−0.061	−0.042	0.115^**^	0.391^**^	0.073	1											
Commercial (X_9_)	−0.030^**^	−0.036	−0.021	−0.008	0.048	−0.060	−0.136^**^	0.022	−0.136^**^	1									
Family type (X_10_)	0.064	0.020	−0.073	0.050	0.104^**^	0.027	−0.135^**^	0.057	−0.135^**^	0.115^**^	1							
House Type (X_11_)	−0.092^*^	0.029	0.029	0.019	0.027	−0.048	0.022	0.030	0.022	−0.144^**^	−0.057	1					
Vaccination (X_12_)	−0.275^***^	−0.093^*^	0.030	−0.056	0.016	0.069	−0.101^**^	0.031	−0.101^**^	0.118^**^	0.061	0.081^*^	1			
Information (X_13_)	−0.178^***^	−0.001	−0.091^*^	0.009	−0.058	−0.148^**^	−0.152^**^	−0.103^**^	−0.152^**^	−0.018	0.126^**^	0.082^*^	0.197^**^	1	
Training (X_14_)	0.322^**^	−0.140^**^	0.243^**^	−0.103^**^	0.031	0.121^**^	−0.100^*^	0.068	−0.100^*^	0.115^**^	0.099^*^	0.076	0.245^**^	0.042	1

^*^significance at 10%, ^**^significance at 5% and ^***^significance at 1.

### 3.5 Factors influencing the mortality ratio

The results mentioned in Table 5 indicate that several independent variables have a significant impact on the ratio of mortality. Results for education showed a negative association (co-efficient= −0.072) with mortality loss and was significant at value of *p*<0.01. The variables with significant co-efficient values and value of *p* at the 0.05 level or below is education, experience, household income per month, family members, domestic-commercial, commercial, vaccination, and information. Education, experience, household income per month, family members, and vaccination are negatively related to the ratio of mortality loss, indicating that as these factors increase, mortality ratio decreases. Furthermore, domestic-commercial and commercial have a strong negative association with mortality ratio. Information is also negatively related to mortality ratio with a co-efficient of −0.286 and value of *p*<0.01, indicating that increased access to health-related information may be associated with reduced mortality ratio. Household size, house types and training do not appear to significantly impact the mortality ratio, as their value of *p* are greater than 0.10. The log pseudo-likelihood and Wald Chi^2^ values suggest that the model as a whole is a good fit for the data.

**Table 5:** Factors affecting the mortality losses from LSD.

Independent variables	Co-efficient	Robust St. error	Value of *p*
Age	0.0001	0.002	0.815
Education	−0.072	0.016	0.000^***^
Experience	−0.011	0.003	0.000^***^
Household income per month	−0.000	6.75×10^−6^	0.001^***^
Household size	−0.008	0.008	0.333
Domestic	0.132	0.114	0.248
Domestic-commercial	−0.283	0.110	0.001^***^
Commercial	−1.369	0.088	0000^***^
House type	−0.069	0.056	0.215
Vaccination	−0.335	0.053	0.000^***^
Information	−0.286	0.103	0.006^***^
Training	0.067	0.095	0.484
Constant	2.998	0.286	0.000^***^
Log pseudo likelihood	−144.38		
Wald Chi^2^	849.		
Value of *p*	0.000		
*n*	406		

^***^significance at 1%.

### 3.6 Marginal effects (ME)

Marginal effects refer to the rate of change of the regression equation for each variable within the model, calculated for every individual data point. The results show that the education and experience of the respondents have significant negative marginal effects on mortality ratio, indicating that higher education and more experience are associated with lower mortality ratio. A unit increase in education and experience decreases the likelihood of the ratio of mortality ratio by 9.5 and 7.3%, respectively (Table 6). The household income per month has a significant (value of *p*<0.01) negative marginal effect, suggesting that a unit higher income is associated with lower mortality ratio by 10.1%. Household size and animal for domestic purpose do not have significant marginal effects. Domestic-commercial and commercial farming have significant negative marginal effects on mortality ratio, indicating that commercial farming practices are associated with lower mortality ratio. Vaccination has significant marginal effects (−0.033) on mortality ratio. This implies that vaccination has decreased mortality ratio by 3.3%. The information source has a significant negative marginal effect (−0.039) on mortality ratio, suggesting that access to information is associated with lower mortality ratio.

**Table 6:** Marginal effects.

Independent variables	ME	Delta-method SD	Value of *p*
Age	0.006	0.024	0.814
Education	−0.095	0.022	0.000^***^
Experience	−0.073	0.019	0.000^***^
Household income per month	−0.101	3.11×10^−2^	0.001^**^
Household size	−0.016	0.017	0.330
Domestic	0.006	0.006	0.258
Domestic-commercial	−0.040	0.016	0.011^**^
Commercial	−0.036	0.002	0.000^***^
House type	−0.088	0.008	0.223
Vaccination	−0.033	0.006	0.000^***^
Information	−0.039	0.015	0.008^***^
Training	0.016	0.022	0.479

^**^significance at 5% and ^***^significance at 1%.

## 4 Discussion

The livestock industry in Pakistan was highly vulnerable to the economic effects of LSD (8). The output of infected animals often decreased, and in extreme circumstances, the mortality rate was high.

The findings of the study showed that LSD has emerged as a significant economic threat to the livestock sector in Pakistan. The study findings showed that mortality ratio 0.48, which was very high like many other developing countries such as Kumar and Tripathi (42) and Parvin et al. (43) reported up to 5% in India and Bangladesh. Moreover, the findings showed that LSD affected animals’ health and productivity and has significant economic impacts at the farmers’ households. The outbreak of lumpy skin disease resulted a significant financial loss for farmers due to the direct and indirect costs associated with the disease. These costs include treatment and management expenses. Kiplagat et al. (37) have assessed and found that most losses were direct losses resulting from reduced production. In addition to the direct losses, the disease caused indirect losses in the form of wage loss of the farmers who were engaged in taking care of animals during the disease. The reduction in milk production can be particularly damaging for smallholder farmers who rely on milk sales as a source of income which was also the case in Thailand due to LSD (24). In addition, the findings showed that the mean value for mortality losses in indigenous cattle was low compared to that of exotic cows. According to the findings of this study, herds of indigenous cattle were less susceptible to disease than those of exotic breeds. These results are similar to that of Khalafalla et al. (44) and Kiplagat et al. (37) from Sudan and Kenya respectively, that indigenous cows are less susceptible to diseases than exotic cows. The mean value for total mortality losses in PKR was quite high, indicating that the economic losses due to mortality in the livestock industry are substantial. The high economic losses due to mortality indicated that efforts to reduce the mortality rate in the livestock industry, vaccination and treatment were necessary to tackle the problem of high mortality ratio. However, vaccination and treatment were not for free and caused additional financial burdens on the farm households. For instance, vaccination and treatment costs per animal were PKR. 175.84 and 6869.69 respectively, indicated the need for affordable vaccines and treatment of animals (45). The disease has decreased farmers’ income, which might have a ripple effect on the local economy, as farmers might have less disposable income to spend on other goods and services (46). Therefore, the findings call for measures to improve the health and welfare of livestock, reduce mortality losses, and ensure the economic sustainability of the livestock dependent households.

The findings further revealed the farmers’ perceptions about the reasons for LSD. For instance, climate change can impacts the incidence and spread of lumpy skin disease through various direct and indirect mechanisms (47). Therefore, it is important to consider the potential impacts of climate change when designing and implementing measures to prevent and control the spread of LSD. The farmers also stated that high growth of population and urbanization were the important reasons for the high spread of LSD. This is because as the populations grow, cities and towns expand, that being so encroaching on rural areas and natural habitats. According to Adla et al. (48), urbanization leads to changes in land use, deforestation, and the fragmentation of ecosystems, which can alter the distribution and abundance of biting flies, the main vectors of LSD.

Furthermore, the results suggested that education, experience, household income per month, family members, vaccination, keeping animals for domestic-commercial, commercial purposes, and information access significantly impacted the mortality ratio. The negative association between education and mortality ratio suggests that educated people were more likely to have access to healthcare services and were able to understand health-related information, such as how to maintain their animals’ health. The same results were reported by Islam (35) from Bangladesh and stated that educated farmers were more involved in-time vaccination of their animals against LSD. Besides education, the negative relationship between experience and mortality loss is also noteworthy, as it indicates that people who have faced similar health crises in the past, such as an LSD epidemic, may be more equipped to deal with it. They may have acquired the wherewithal to lessen the severity of the disease’s effects, hence decreasing mortality rates. Morgenstern et al. (36) revealed that experienced farmers vaccinated their animals more than inexperienced farmers. It also suggested that experience can contribute to better disease management and prevention efforts within the farming community.

The findings related to monthly households’ income indicated that economic factors are important in reducing mortality ratio. Higher household income can provide better access to veterinary care, preventative measures such as vaccinations and higher-quality nutrition for animals, all of which can reduce mortality rates. In addition, higher income may allow families to invest in better housing and sanitation for their animals, which can also contribute to better health outcomes. Our findings for income and animal health are in agreement with the findings of Card et al. (49), who reported from North America that the disparities extend beyond the traditional concerns of animal welfare, such as individual care and treatment. Instead, they are correlated with the financial resources available to animal caregivers. Inadequate income can hinder the ability of animal caregivers to provide essential medical care, nutrition, and other aspects of animal husbandry, potentially resulting disparities in animal health and welfare. Moreover, the findings showed that individuals who own or manage domestic-commercial animals have lower mortality ratio than those who keep animals for domestic purposes only. This could imply that domestic-commercial animals’ owners or managers have a greater understanding and investment in animal welfare and health. Likewise, if the animals were pure commercial, they were less exposed to diseases. In this connection, McGlone (38) discovered that farm animal welfare is a critical concern particularly in industrialized countries. In addition, the findings showed that individuals who vaccinated their animals had lower mortality ratio. This could imply that vaccination is an important factor in promoting animal’s health. Molla et al. (39) reported from Ethiopia that LSD prevention and control strategies are based on regular vaccination. The access to information sources such as extension services were used as a variable in the model, suggesting that individuals who had accessed extension services bear less loss of animals’ mortality. Moreover, in the study area the farmers also used TV, Radio, friends, social media and mobile phones for accessing information. Habiyaremye et al. (45) showed that farmers in South Africa used phones, TV and radio as sources of information for animals’ better healthcare. This highlighted the potential for media campaigns and outreach programs to educate the public and raise awareness about animal’s welfare issues. This could imply that access to information is important in promoting animals’ welfare as LSD is a highly contagious viral disease that affects mostly cows and buffalos. Whereas, can cause significant economic losses in the livestock sector, where there are limited extension services, and contribute to low agricultural productivity in developing nations, including Pakistan. The study highlighted the need for greater awareness and education on LSD and adaptation among farmers in the study area.

## 5 Limitations and strengths of the study

The study was conducted in only one district of Khyber Pakhtunkhwa in Pakistan, which may limit the generalizability of the findings to other parts of the province or country. The data collected in the study might have been influenced by self-reporting bias, as participants might not have been completely confident about their responses. Moreover, it is cross-sectional study and we could not collect longitudinal data, which would provide more insight into the long-term effects of LSD on livestock productivity and farmers’ income. However, the study is based on primary and original data from the farmers, which can be used as a baseline study to work in future. The study uncovered that LSD has had a significant monetary impact on farmers’ households. It provided numerical data on how the disease has affected animals’ welfare, farm output, and farmers’ income.

## 6 Conclusion

The study highlighted the significant economic impact of LSD on the livestock sector in Pakistan. The disease has caused various direct and indirect losses.

### 6.1 Main findings

The reduction in milk production was particularly damaging for smallholder farmers who relied on milk sales as a source of income. Moreover, the study showed that mortality losses due to LSD were a significant problem in the livestock industry, with exotic cows particularly vulnerable. The economic losses incurred by livestock farmers due to diseases have ripple effects on the local economy. The study also revealed the farmers’ perceptions about the reasons for LSD. The responses suggested that climate change and more population and urbanization contributed to the high spread of LSD. Moreover, the study explored the factors that determine mortality ratio from LSD, such as education, experience, household income per month, vaccination, domestic-commercial animal, commercial animals, and access to extension services for information.

### 6.2 Policy implications

To mitigate the economic impacts of lumpy skin disease, it is crucial to implement effective prevention and control measures. This includes free vaccination programs, prompt detection and isolation of infected animals. Additionally, providing farmers with information and education on disease prevention and control can help reduce the spread of the disease and minimize economic losses. By implementing these measures, farmers can better protect their livestock, their livelihoods, and the wider community from the devastating economic impacts of LSD.

### 6.3 Future recommendations

In order to enhance the validity of the results, it is recommended that future investigations undertake similar studies in various parts of Pakistan. This will provide a more comprehensive understanding of the effects of LSD on livestock and the financial status of farmers in various geographical and socio-economic settings. Future research should seek to acquire longitudinal data to track the long-term effects of LSD and estimate the mortality rate This would shed light on the disease’s long-term effects on livestock productivity and farmers’ income.

## Data availability statement

The original contributions presented in the study are included in the article/supplementary material, further inquiries can be directed to the corresponding author.

## Ethics statement

The studies involving humans were approved by Research Ethics Committee AWKUM. The studies were conducted in accordance with the local legislation and institutional requirements. The participants provided their written informed consent to participate in this study.

## Author contributions

All authors listed have made a substantial, direct, and intellectual contribution to the work and approved it for publication.

## Footnotes

^1^Joint family means all family members living together, and nuclear is for living with wife and kids.

^2^Kacha house means the house made from wood and straws while pacca house are made from breaks and cement.

^3^According the SBP data, 1 USD= PKR 283.7919.
